# A realist review of educational interventions to improve the delivery of nutrition care by doctors and future doctors

**DOI:** 10.1186/2046-4053-3-148

**Published:** 2014-12-20

**Authors:** Victor Mogre, Albert Scherpbier, Tim Dornan, Fred Stevens, Paul Armah Aryee, Mary Gemma Cherry

**Affiliations:** Department of Human Biology, School of Medicine and Health Sciences, University for Development Studies, Tamale, Ghana; School of Health Professions Education, Maastricht University, Maastricht, The Netherlands; Department of Health Services Research, Institute of Psychology, Health and Society, University of Liverpool, Liverpool, UK

**Keywords:** Realist review, Realist synthesis, Educational interventions, Nutrition care, Future doctors, Improve, Delivery

## Abstract

**Background:**

Dietary interventions are considered an important aspect of clinical practice, more so in the face of the rising prevalence of obesity, diabetes and cardiovascular diseases globally. Routinely, most doctors do not provide such intervention to their patients, and several barriers, present during both training and clinical practice, have been identified. Educational interventions to improve nutrition care competencies and delivery have been implemented but with variable success, probably, due to the complex nature of such interventions. Using traditional methods only to investigate whether interventions are effective or not could not provide appropriate lessons. It is therefore pertinent to conduct a realist review that investigates how the interventions work. This realist review aims at determining what sort of educational interventions work, how, for whom, and in what circumstances, to improve the delivery of nutrition care by doctors and future doctors.

**Methods/design:**

This realist review will be conducted according to Pawson’s five practical steps for conducting a realist review: (1) clarifying the scope of the review, (2) determining the search strategy, including adopting broad inclusion/exclusion criteria and purposive snowballing techniques, (3) ensuring proper article selection and study quality assessment using multiple methods, (4) extracting and organising data through the process of note taking, annotation and conceptualization and (5) synthesising the evidence and drawing conclusions through a process of reasoning. This realist review protocol has not been registered in any database before now.

**Discussion:**

Findings will be reported according to the publication criteria outlined by the realist and meta-narrative evidence synthesis (RAMESES) group.

## Background

Dietary interventions, such as discussing weight loss and providing dietary counselling to obese patients, are an important part of clinical practice. Most doctors, however, do not routinely provide them [1, 2]. There are several reasons for this, including lack of time and lack of confidence that patients will comply [1]. In addition, doctors lack confidence, motivation, skills and knowledge, which results from inadequate training during medical school [1, 3–5]. Fifty percent of graduating US medical students report that the time devoted to nutrition education in medical school and the nutritional content of their curricula are inadequate [6].

On the other hand, doctors have generally positive views on the role of nutrition in clinical practice [1, 2] and would give nutrition care (for example dietary counselling) to their patients if it were not for the various barriers outlined above [1]. Those barriers are present during both training and practice. Educational interventions to improve medical students’ and doctors’ nutrition care competencies and delivery have been undertaken during training and in practice with variable success [7–9]. The delivery of nutrition care by doctors is therefore still in need of appropriate models [7–9]. It is important to identify key components of effective educational interventions and which educational interventions and strategies should be used in which settings [10].

Reviews on the effectiveness of educational interventions to improve the nutrition care competencies and delivery of doctors are limited. In our literature search, we came across only one review that considered the effectiveness of nutrition training programs to improve health workers’ (including doctors) nutrition knowledge and competence to manage child under nutrition [11]. The review by Sunguya et al. followed a traditional systematic review process. To the best of our knowledge, no review be it traditional or realist, has considered the effectiveness of educational interventions to improve the nutrition care competencies of future doctors only and/or both doctors and future doctors. This is probably due to the complex nature of such interventions. As such there is paucity of data exploring how educational interventions might work most effectively, how, why (the mechanisms beneath) and under what circumstances to improve the delivery of nutrition care by future doctors and doctors. This review aims to fill these gaps.

The strength of systematic reviews is that they can measure the effectiveness of interventions in terms of their effect sizes. They aspire to be relatively free of context effects, which confound the relationship between an intervention and its outcome. In the words of Cook et al. [12], classical systematic reviews are “justification research”. Systematic review was introduced in the biomedical domain to help doctors choose between different treatments [13, 14]. There is a preference within the education domain, however, for “clarification research”, which seeks not so much to find out whether an intervention works but as to how and why it works [12]. From that perspective, a systematic review methodology has shortcomings: notably, that the drive to minimise bias oversimplifies the inherent complexity of interventions and their inherently contextual nature [15, 16]. This may result in misleading oversimplification and an inability to account for the inescapable complexity of interactions between conditions, mechanisms and outcomes [17].

In this realist review, we conceptualise educational interventions and strategies to improve the delivery of nutrition care as complex ones [18], which involve multiple actors (teachers, learners, patients, health care providers, etc.) operating at different levels, the artefacts they use and the material environments in which they work [17]. In a non-linear fashion, these components interact to produce context-dependent outcomes.

The broad purpose of this realist review is to determine what sort of educational interventions work, how, for whom, why and in what circumstances to improve the delivery of nutrition care by future doctors and doctors. Considering educational interventions to improve the delivery of nutrition care by future doctors as well as those to improve the delivery of nutrition by doctors, will grant us the opportunity to identify mechanisms and contextual factors that influences those educational interventions during training and in practice. This will provide curriculum leaders and policy makers with evidence, which will help them, design and implement educational interventions that are appropriate to the settings, people and educational goals they are aiming to achieve. Specifically, it sets out to answer the following review questions:

### Review questions

1. What learning outcomes do future doctors and doctors need to attain in order to deliver nutrition care to patients?
2. What mechanisms and contextual factors lead to the attainment of those outcomes?
3. How do those mechanisms and contextual factors interplay to produce those outcomes?
4. How could undergraduate educational interventions support an optimal interplay of those factors?

## Methods/design

### Study design

As informed by the above research questions, the realist review will follow principles outlined by Pawson [16]. These principles emphasise the search for evidence supporting complex interventions and providing explanations for why they may or may not work, how and in what contexts [16, 19]. Fundamentally, a realist review is concerned with developing and refining theory [16, 20, 21], accounting for context as well as outcomes in the process of systematically, iteratively and transparently synthesizing relevant literature [22, 23]. It aims to unpack a context-mechanism-outcome (CMO) relationship, explaining examples of success, failure and various eventualities in between [24]. The theoretical explanations that result from this process are further configured and refined to construct an “overarching” realist programme theory [25] The key product of this review, therefore, is the building and refinement of a programme theory which enables us to achieve a level of abstraction needed to understand the diversity of outcomes produced in different contexts [26]. The programme theory of an intervention is the specification of how and why the programme or intervention is conceived to cause its intended outcomes, taking into consideration the active mechanisms and contexts [26]. The theory is built based on available evidence and refined throughout the study.

The review will be conducted following Pawson’s five practical steps for conducting a realist review [20]. These steps include: 1) clarifying the scope of the review; (2) determining the search strategy; (3) ensuring proper article selection and study quality assessment; (4) extracting and organising data and (5) synthesising the evidence and drawing conclusions through a process of reasoning [16, 20].

### Theory initiation

An important component and obviously the end goal of the realist review process is the development or refinement of a programme theory(ies). As suggested by Pawson [16, 20], we will tap into stakeholders and experts to help in this regard. Exploration of key theories has already begun through on-going conversations within the review team and discussions with health professions educational experts, teachers, students, physicians, among others. A concept map is being developed of ideas, views and opinions on why educational interventions work, who they work for, in what circumstances and why. This will be explored in the literature searches “to identify the theories, hunches, expectations, and the rationalizations” [16] for why educational interventions may or may not work to improve the delivery of nutrition care [16, 20] by future doctors and doctors. This exercise is aimed at identifying “a range of theories and explanations for how educational interventions are supposed to work (and for whom), when they do work, when they don’t achieve the desired change in nutrition care delivery, why they are not effective in this and why they are not being used” [27]. These initial on-going processes have resulted in the following candidate programme theories which will be continuously refined and strengthened as they transform (or emerging theories arises) and finalised through the testing of their validity during the realist review process.

### Candidate programme theories

Riding on the back of existing substantive theories of expertise development [28–31], the delivery of nutrition care is a complex skill that is influenced by the following: (1) nutrition care competency, (2) the individual motivation of the future doctor/doctor, self-regulation and metacognition and (3) the workplace setting (e.g. the hospital). The delivery of nutrition care by doctors is a capability that is influenced by cognitive and affective processes (knowledge, skills, attitudes, self-efficacy, etc.) of the individual as well as the social, cultural and physical environmental context in which the behaviour is executed.

Nutrition care competency is a broad concept that encompasses knowledge, skills and attitudes. It is the capability to apply or use nutrition knowledge and skills/abilities needed to successfully perform the task of delivering nutrition care to patients in a defined work setting (e.g. the hospital). We recognise that the delivery of nutrition care is influenced by the availability of adequate competency which is the outcome of a learning process that is influenced by factors within the academic environment relating to the quantity, quality and nutrition content in the curriculum as well as the teaching and learning methods employed and the reinforcement experienced by the individual and by others. It is our hypothesis that doctors will deliver nutrition care by a process of mimesis [32], which means building on and making personal behaviours and attributes demonstrated by colleagues (e.g. course mates) and superiors (e.g. specialist, consultants, clinical teachers, etc.) delivering nutrition care.

The delivery of nutrition care is also influenced by the doctor’s own thoughts, self-beliefs and their interpretation of the environment relating to Bandura’s social cognitive theory [33]. Doctors are more likely to deliver nutrition care to their patients if they have high self-efficacy for it and less likely to deliver nutrition care if they do not feel self-efficacious. In accordance with the social cognitive theory, knowledge (cognitive), behavioural, personal and environmental factors interact to determine motivation and behaviour [34].

Even though nutrition care competency is an important determinant of the delivery of nutrition care, it does not alone predict the delivery of nutrition care for the promotion of health and well-being [3]. The delivery of nutrition care is also influenced by the social environment (the workplace setting). We assume that the delivery of nutrition care by doctors is a behaviour demonstrated in the social context of the workplace, which is influenced by observing and modelling the behaviours, attitudes and emotional reactions of others (e.g. superiors) [35]. It is also influenced by the structural determinants of behaviours such as the nature of the workplace setting (e.g. hospital/community, emergency/paediatric/general ward), job descriptions/role, time and availability of other staff to undertake particular roles.

**Figure 1:**
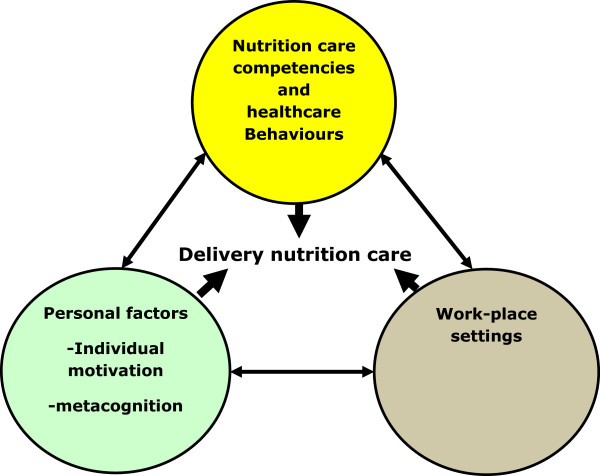
**Theoretical model.** Adapted from Wood and Bandura’s Triadic Reciprocal Determinism (Wood and Bandura [36]).

The interaction of these three factors (nutrition care competencies, the individual’s motivation and workplace setting) can be likened to Wood and Bandura’s [36] Triadic Reciprocal Determinism model, in that the on-going functioning of nutrition care delivery is the product of a continuous interaction between cognitive, behavioural and social-environmental factors. Therefore, our candidate programme theory is that the delivery of nutrition care is a complex process that is influenced not only by cognitive skills, concepts and abstract rules but also by the individual’s motivation as well as the social environment within which nutrition care is delivered (see Figure 1).

### Determining the search strategy

As is customary in a realist review, broad inclusion/exclusion criteria will be employed to ensure that the process of programme theory development takes in the widest range of evidence [20].

### Inclusion criteria

#### Types of data

In keeping with the nature of a realist review, both quantitative and qualitative studies will be included. Both published and grey literature (such as websites, reports of international organizations as well as dissertations and theses) will be consulted.

#### Study design

Randomised control trials (RCTs), cluster RCTs, quasi-experimental studies, pre-post-intervention longitudinal studies (with or without comparison groups), non-interventional qualitative studies, cross-sectional studies and case reports will be included.

#### Participants

Studies including healthcare professions students (including medicine) will be included. In addition, those having doctors as learners will also be included.

#### Search limitations

The search will be limited to a 20-year-publication period (1994–2014), in order to restrict the research to relatively recent evidence whilst ensuring sufficient evidence.

#### Study focus

Studies with nutrition training/educational interventions will be included. Non-interventional studies will also be considered if they provide information about how conditions, mechanisms and outcomes interrelate.

#### Outcome measures

All studies that have nutrition competencies and behaviours and/or delivery of nutrition care as the outcome measure will be considered.

### Exclusion criteria

Studies will be excluded if they are not relevant to nutrition care competencies and/or delivery or if they are purely anecdotal.

### Search process

A combination of MeSH/thesauri and free text terms will be employed in the search strategy. Truncation and appropriate Boolean operators will be employed. Scoping searches will be conducted to refine the search terms further. The following search terms will be employed.

The terms will be broad and will be grouped into participants, outcomes and conditions as shown below.

-  
*Participants.* Medical students; health professions students; general practitioners; doctors; primary care physicians.
-  
*Outcomes*. Diet counselling, nutrition education; eating behaviours; preventive medicine; nutritional management; lifestyle modification; nutrition therapy
-  
*Conditions*. Curriculum; integrated; training; evaluation; assessment; impact; learning; undergraduate; medical education; capacity; capacity building

Searches will be conducted across the following online databases: MEDLINE, CINAHL, ERIC, Embase, PsycINFO, Sociological Abstracts, Web of Science and Google Scholar and any other database related to medical education. Grey literature and thesis databases will also be consulted. Recognizing the limitations of a traditional search strategy [37], an expert-directed search using a multiple-search strategy approach will be incorporated. As stated by Kastner, a multiple-search strategy approach seeks to explore and contextualise the intervention in multiple settings resulting in the strategy being iterative and interactive [15, 16]. This process is flexible and allows us the benefit of capitalising on unanticipated findings [27]. Experts will be identified through snowball searching. These experts will then be consulted to help us look for and find potentially relevant literature and concepts. The reference list of relevant articles from the core articles will be used to find potentially relevant studies from bibliographic references.

As expected in interpretive research like this realist review, the search for new studies will end at the point of theoretical saturation, a point at which the evidence available meets the theoretical need or answers the research questions [16, 20, 38]. In consonance with the suggestion of Pawson [16, 20], the test of saturation will be obtained iteratively. At each stage or cycle, the latest addition of literature would be assessed to determine whether something new has been added to our understanding of the programme theory.

A reviewer will ensure that all relevant journals and studies are adequately indexed in the databases selected. In addition, members of the research team will review the final list of studies to identify and correct obvious omissions using their knowledge. Potentially eligible studies based on the inclusion and exclusion criteria described above will be obtained in full text and downloaded into Reference Manager Professional Version 11.0. The content of the file will be backed up to a secured server.

**Figure 2:**
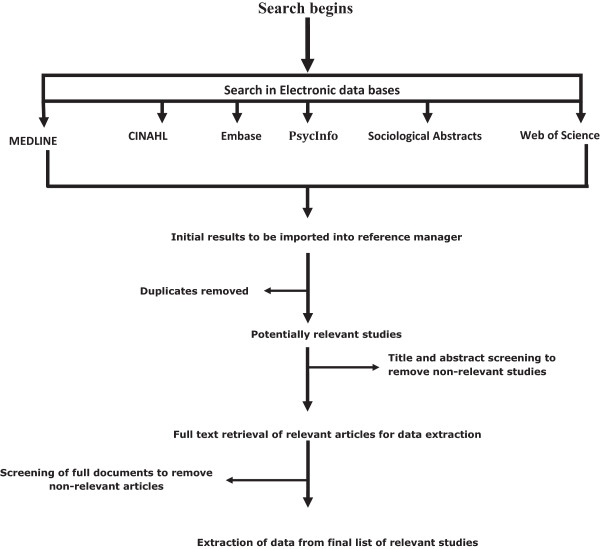
**Search strategy.**

Shown in Figure 2 is a diagram of the proposed search strategy.

### Selection of studies and quality assessment

Unlike systematic reviews, the quality assessment of studies will not be based on the hierarchy of evidence [20]. Pawson argues that multiple methods of assessment should be employed as a measure of quality in realist synthesis in order to “illuminate the richest picture” [20, 27]. The heuristic approach which will be employed is one where the value of a study is assessed by how much it is able to enrich the programme theory. It should answer the question: “Does the research address the theory under investigation?” Usually, the unit of assessment is not the study itself but sections of related theory and evidence [16].

As an initial step, two research team members in consultation with the wider group will independently screen the title, abstract and subject headings of the searched studies against the initial inclusion and exclusion criteria (which will evolve during the process of the review). This initial process by the two reviewers will afford us the opportunity to maintain a certain level of rigour (i.e. refinement and proper interpretation of the inclusion and exclusion criteria). It is also a valuable platform for reflexive discussion that will enable informed decisions among reviewers for identifying relevant data [39]. As we anticipate a high volume of potentially relevant studies, provided there is high level of agreement between the two reviewers, this initial process may reduce the number of studies that will need to be reviewed further.

A key step of our review is the process of determining rigour. Pawson describes the process of determining “rigour” “as whether a particular inference drawn by the authors has sufficient evidence to make a methodologically credible contribution to the test of a theory” and to apply “judgement” to supplement formal critical appraisal checklists (if they are used) [20]. In the absence of technical procedures to describe “judgements”, we would adopt an approach that will use rigour (e.g. methodological rigour) as a supporting/complementary/mediating tool rather than the only means to determine the quality of a study. This will grant us the opportunity to include studies that best inform our purpose (e.g. for studies that have the same concepts but with differing methodological rigour or to adjudicate between studies that have similar methodologies but conflicting results). Judgement will be used to resolve conflicts or disagreements between reviewers by determining how the results of the study(ies) could be applied to the context of using educational interventions to improve the delivery of nutrition care by future doctors and doctors. This will be done through discussions among members of the review team. In order not to exclude studies based on methodological rigour, studies will be selected and reviewed based on what new knowledge (which could come from any part of the study including introduction, methodology, results and discussion sections) it brings on board for the development and refinement of the programme theory. Furthermore, the meaning and value of rigour will be defined, examined and documented for each selected study to ensure transparency. This will include decisions about rigour and relevance (the contribution the study either as a whole or a section makes to the review). If need be and appropriate, appraisal tools may also be used to determine rigour. Similar to systematic reviews, the importance of transparency in a realist review is to ensure the validity, reliability and verifiability of findings and conclusions [27, 40].

### Data extraction and organisation

In meeting the requirements of this theory-driven approach, our review will include all potentially relevant studies that add to the development of our theory [41]. Data “extraction” and organisation will be done through a process of note taking, annotation and conceptualization. In addition, our conceptualization of the pertinent processes that constitute the implementation of educational interventions to improve the delivery of nutrition care by future doctors and doctors will be refined.

In order to build and refine our programme theory/theories, a set of basic information from the selected studies will be collected into a standard data extraction form by the first author. To ensure accuracy, the initial results of this process will be cross-checked by another reviewer; emphasis on the data extraction fields involving qualitative data or key information (e.g. study design). Disagreements will be resolved by involving other members of the review team. Each study will be read and re-read to capture themes and concepts that might contribute to the building and refinement of the theory. The entire data extraction process will involve critical discussion between the two reviewers (first author and the cross-checking reviewer) and the wider team to ensure that data are used to develop a line of argument(s) that feeds into the final synthesis stage rather than simply classifying them.

### Data analysis and synthesis

In a realist review, data analysis and synthesis are iterative processes that may occur sequential or in parallel [19]. Data analysis in a realist review aims to analyse data using realist concepts employing a philosophical “eye” [19]. Realism is based on a generative explanation for causation; outcomes (O) are generated by relevant mechanisms (M) activated in a context (C) resulting in a CMO configuration. The key task is to refine a programme theory that in general explains determining what works, for whom, in what circumstances, in what respects and why [42]. Pawson et al. suggest that synthesis partly consists of processes of reasoning including juxtaposing, reconciling, adjudicating, consolidating and situating all sources of evidence that helps to refine the programme theory [15, 16]. We believe that these processes are insufficient on their own as they do not provide a complete description of synthesis of the data. To cater for these limitations, we include the principles of thematic analysis to analyse and synthesize our data. It is an appropriate compromise that combines the principles of meta-ethnography and grounded theory [43]. Reciprocal “translation” a feature of meta-ethnography is realised through the coding of findings to develop descriptive and analytical themes. As a feature of grounded theory adapted into thematic analysis, the process is inductive and the development of themes is through the process of constant comparison [43]. Recognising the basic principles of realist synthesis and thematic analysis, we intend to analyse and synthesize our data according to the following steps, a modification of the approach proposed by Rycroft-Malone et al. [42]:

- Extracted data will be summarised and organised into theory areas and review questions related tables.
- Individual reviewers will independently theme the extracted data using “free codes”.
- Identified themes will be collated and discussed by members of the review team. This will provide an opportunity to discuss and resolve differences that may arise.
- Through discussions among members of the review team, chains of inferences will be identified. A chain of inference refers to linkages between the various themes that have been identified [42] as well as linking them to the primary data that generated the themes. This will be done by keeping track of the primary data that generated the themes. Proceeding, connections will be made among chains of inferences. This will be an iterative process in which chains of inferences will be linked to each other as well as to the primary source of evidence.
- Hypotheses will be developed based on the chains of inferences [42]. Conceptually, themes will be linked to chains of inference, which will be linked to a hypothesis. All included studies will be linked explicitly to chains of inferences and hypotheses. The output of the previous steps will result in a cumulative picture of potential mechanisms, contexts and outcomes chains [42]. The generated hypotheses will then be used to either form new programme theories or refine them. Narrative synthesis will be developed around each programme theory(ies), summarizing the nature of the context, mechanism and outcome links and the characteristics of the primary evidence underpinning them (individual included studies) [42]. These will be presented using text, summary tables, a logic model and where appropriate graphics [44].

The origins of the inferences, the hypothesis and the subsequent programme theories and their specific sources, on which they are grounded, will be documented and justified. The reasoning processes explaining how they are rooted in the primary evidence will be documented to ensure transparency. For example, how an included study contributed to a theme, which resulted in the formation of a chain of inference, subsequently resulting in a hypothesis and finally contributing to a programme theory. We will also document the inferential shifts that occur during the reasoning process to engage the evidence.

This realist review protocol has not been registered in any database.

## Discussion

### Formation of a review advisory group

In order to meet the requirement of involving stakeholders in the realist review, a review advisory group will be formed. The review advisory group will be comprised of all members of this research team and will also include teachers, students, curriculum experts, doctors, among others. The composition of the group will depend on the stage of the realist review. The advisory review group will assist in building programme theories. The advisory review group will provide a “reality check” on the clarity and explanatory strength of identified and selected theories.

### Importance of the review

The findings of the review may be useful to teachers, students, researchers in medical education as well as policy and decision makers in health professions education and to patients in the long term. As indicated in the introduction section of this review protocol, the delivery of nutrition care by doctors is still an area in need of an appropriate model. This review will help in the realization of such models by identifying the context and mechanisms under which such models operate successfully. Also this review forms part of a PhD project aiming at developing an intervention to improve the nutrition care competencies and delivery of health care providers. This initial review will help explain the intrinsic reasons for why and under what conditions educational interventions improve nutrition care competencies during training and delivery in practice. This is an important first step toward a better understanding of characteristics of educational interventions to help improve nutrition care competencies and delivery.

A realist review of literature is considered a relatively new methodology and few researchers in health professions education have used it. Obviously, conceptual and methodological challenges might have contributed to this situation [38]. A secondary importance of this realist review is to facilitate its adoption by other researchers in health professions education. This will be realised through the publication of a methods paper through this review protocol and the findings of the review.

### Reporting and dissemination of findings

Our findings will be reported according to the publication criteria outlined by the realist and meta-narrative evidence synthesis (RAMESES) group [19]. The research questions will be explored, answered and reported in a language that is acceptable to all stakeholders. The findings of the review will be reported through two activities. Firstly, we will report the findings in an international journal specializing in medical education, implementation science or public health or medical journals. Secondly, the results of the review will be presented in international conferences such as The NETWORK-Towards Unity for Health annual conference, AMEE and among others.

## Abbreviations

RAMESES: Realist and meta-narrative evidence synthesis
The NETWORK: TUFH: The Network Towards Unity for Health
AMEE: The Association for Medical Education in Europe.
